# (2*E*,6*E*)-2,6-Bis(2,4,5-trimethoxy­benzyl­idene)cyclo­hexa­none

**DOI:** 10.1107/S1600536810005192

**Published:** 2010-02-13

**Authors:** Tara Shahani, Hoong-Kun Fun, G. L. Balaji, V. Vijayakumar, S. Sarveswari

**Affiliations:** a X-ray Crystallography Unit, School of Physics, Universiti Sains Malaysia, 11800 USM, Penang, Malaysia; b Organic Chemistry Division, School of Advanced Sciences, VIT University, Vellore-632 014, India

## Abstract

In the title compound, C_26_H_30_O_7_, one atom in the cyclo­hexa­none ring is disordered over two positions with a site-occupancy ratio of 0.871 (6):0.129 (6). The dihedral angles formed between the mean plane through the six C atoms of the major component of the cyclo­hexa­none ring and two benzene rings are 35.09 (10) and 34.21 (10)°; the corresponding angles for the minor component are 20.1 (2) and 19.5 (2)°. Both the major and minor disordered components of the cyclo­hexa­none ring adopt half-boat conformations. In the crystal packing, inter­molecular C—H⋯O hydrogen bonds connect the mol­ecules into a three-dimensional network.

## Related literature

For natural biocides, see: Geiger & Conn (1945 ▶); Marian *et al.* (1947 ▶). For the biological activity and biological properties of chalcones, see: Srivastava *et al.* (1997 ▶); Kuhn & Hensel (1953 ▶); Hosni & Saad (1995 ▶); Ishida *et al.* (1960 ▶); Mehata & Parikh (1978 ▶); Mudaliar & Joshi (1995 ▶). For ring conformations, see: Cremer & Pople (1975 ▶). For hydrogen-bond motifs, see: Bernstein *et al.* (1995 ▶). For bond-length data, see: Allen *et al.* (1987 ▶).
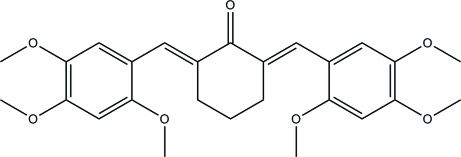


## Experimental

### 

#### Crystal data

C_26_H_30_O_7_
*M* *_r_* = 454.50
Monoclinic, 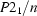

*a* = 9.0943 (1) Å
*b* = 13.4947 (1) Å
*c* = 18.8293 (2) Å
β = 100.449 (1)°
*V* = 2272.50 (4) Å^3^
*Z* = 4
Mo *K*α radiation
μ = 0.10 mm^−1^
*T* = 296 K
0.37 × 0.21 × 0.18 mm

#### Data collection

Bruker SMART APEXII CCD area-detector diffractometer
Absorption correction: multi-scan (*SADABS*; Bruker, 2009 ▶) *T*_min_ = 0.965, *T*_max_ = 0.983
29097 measured reflections
6691 independent reflections
4027 reflections with *I* > 2σ(*I*)
*R*_int_ = 0.036

#### Refinement

*R*[*F* ^2^ > 2σ(*F* ^2^)] = 0.070
*wR*(*F* ^2^) = 0.155
*S* = 1.06
6691 reflections
317 parameters
4 restraints
H-atom parameters constrained
Δρ_max_ = 0.24 e Å^−3^
Δρ_min_ = −0.24 e Å^−3^

### 

Data collection: *APEX2* (Bruker, 2009 ▶); cell refinement: *SAINT* (Bruker, 2009 ▶); data reduction: *SAINT*; program(s) used to solve structure: *SHELXTL* (Sheldrick, 2008 ▶); program(s) used to refine structure: *SHELXTL*; molecular graphics: *SHELXTL*; software used to prepare material for publication: *SHELXTL* and *PLATON* (Spek, 2009 ▶).

## Supplementary Material

Crystal structure: contains datablocks global, I. DOI: 10.1107/S1600536810005192/sj2721sup1.cif

Structure factors: contains datablocks I. DOI: 10.1107/S1600536810005192/sj2721Isup2.hkl

Additional supplementary materials:  crystallographic information; 3D view; checkCIF report

## Figures and Tables

**Table 1:** Hydrogen-bond geometry (Å, °)

*D*—H⋯*A*	*D*—H	H⋯*A*	*D*⋯*A*	*D*—H⋯*A*
C12*A*—H12*B*⋯O1^i^	0.97	2.43	3.364 (3)	160
C25—H25*B*⋯O1^ii^	0.96	2.55	3.450 (3)	156

Symmetry codes: (i) 
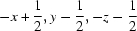
; (ii) 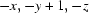.

**Fig. 1.**
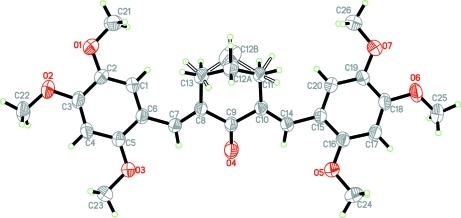
The molecular structure of the title compound, showing 50% probability displacement ellipsoids for the non-hydrogen atoms. Open bonds indicate the minor disordered component.

**Fig. 2.**
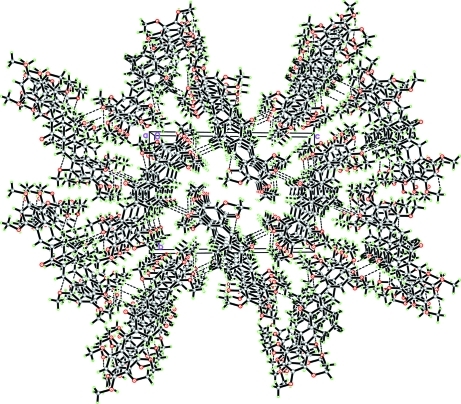
The crystal packing of the title compound, viewed along the a axis, showing the three-dimensional network. Only the major disordered component is shown.

## supplementary crystallographic information

### Comment

The chemistry of chalcones has generated intensive scientific interest due to their biological and industrial applications. Chalcones are natural biocides (Geiger & Conn, 1945; Marian *et al.*, 1947) and are well known intermediates in the synthesis of heterocyclic compounds exhibiting various biological activities (Srivastava *et al.*, 1997; Kuhn & Hensel, 1953). Chalcones and their derivatives possess some interesting biological properties such as antibacterial (Ishida *et al.*, 1960), antifungal (Mehata *et al.*, 1978), insecticidal (Mudaliar & Joshi, 1995), anesthetic (Hosni & Saad, 1995), anti inflammatory, analgesic and ulcerogenic activities.

In the title compound (Fig. 1), the C12 atom is disordered over two positions with a site-occupancy ratio of 0.871 (6):0.129 (6). The dihedral angles formed between major component (C8–C11/C12A/C13) and two benzene rings (C1—C6 and C15—C20) are 35.09 (10) and 34.21 (10)°, and between the minor component (C8–C11/C12B/C13) and two benzene rings are 20.1 (2) and 19.5 (2)°. The major and minor disordered components adopt half-boat conformations (Cremer & Pople, 1975) with puckering of Q = 0.487 (2) Å, Θ = 128.3 (2) ° & φ = 57.8 (3)° and Q = 0.387 (9) Å, Θ = 58.5 (9)° & φ = 242.6 (9)° respectively. In the crystal packing (Fig. 2), intermolecular C12A —H12B···O1 & C25— H25B···O1 hydrogen bonds link the molecules into a 3D network.

### Experimental

A mixture of cyclohexanone (0.5 mmol), 2,4,5-trimethoxybenzaldehyde (4 g) and dry ammonium acetate (0.78 g) was taken in 1:4:2 molar ratio in methanol and heated on water bath till the colour changes to reddish orange, then diethyl ether (50 mmol) was added. The mixture was allowed to stand for 24 h resulting in the formation of needle-shaped crystals. Yield: 60%. *Mp*: 170 °C.

### Refinement

The H atoms bound to C11 and C13 were located in a difference map and then constrained to ride with the parent atoms with *U*_iso_(H) = 1.2*U*_iso_(C). The other H atoms were positioned geometrically. [C—H = 0.9300 to 0.9700 Å]. The C11–C12A, C11–C12B, C13–C12A & C13–C12B distances were restrained with to be equal. The distance between H1A and H13D, H20A and H11C were restrainted to be 2.01 Å.

### Crystal data

**Table:**

C_26_H_30_O_7_	*F*(000) = 968
*M**_r_* = 454.50	*D*_x_ = 1.328 Mg m^−^^3^
Monoclinic, *P*2_1_/*n*	Mo *K*α radiation, λ = 0.71073 Å
Hall symbol: -P 2yn	Cell parameters from 7467 reflections
*a* = 9.0943 (1) Å	θ = 2.7–30.2°
*b* = 13.4947 (1) Å	µ = 0.10 mm^−^^1^
*c* = 18.8293 (2) Å	*T* = 296 K
β = 100.449 (1)°	Block, yellow
*V* = 2272.50 (4) Å^3^	0.37 × 0.21 × 0.18 mm
*Z* = 4	

### Data collection

**Table:**

Bruker SMART APEXII CCD area-detector diffractometer	6691 independent reflections
Radiation source: fine-focus sealed tube	4027 reflections with *I* > 2σ(*I*)
graphite	*R*_int_ = 0.036
φ and ω scans	θ_max_ = 30.2°, θ_min_ = 1.9°
Absorption correction: multi-scan (*SADABS*; Bruker, 2009)	*h* = −12→12
*T*_min_ = 0.965, *T*_max_ = 0.983	*k* = −16→19
29097 measured reflections	*l* = −26→26

### Refinement

**Table:**

Refinement on *F*^2^	Primary atom site location: structure-invariant direct methods
Least-squares matrix: full	Secondary atom site location: difference Fourier map
*R*[*F*^2^ > 2σ(*F*^2^)] = 0.070	Hydrogen site location: inferred from neighbouring sites
*wR*(*F*^2^) = 0.155	H-atom parameters constrained
*S* = 1.06	*w* = 1/[σ^2^(*F*_o_^2^) + (0.0429*P*)^2^ + 1.1706*P*] where *P* = (*F*_o_^2^ + 2*F*_c_^2^)/3
6691 reflections	(Δ/σ)_max_ < 0.001
317 parameters	Δρ_max_ = 0.24 e Å^−^^3^
4 restraints	Δρ_min_ = −0.24 e Å^−^^3^

### Special details

**Table:**

Geometry. All esds (except the esd in the dihedral angle between two l.s. planes) are estimated using the full covariance matrix. The cell esds are taken into account individually in the estimation of esds in distances, angles and torsion angles; correlations between esds in cell parameters are only used when they are defined by crystal symmetry. An approximate (isotropic) treatment of cell esds is used for estimating esds involving l.s. planes.
Refinement. Refinement of *F*^2^ against ALL reflections. The weighted *R*-factor wR and goodness of fit *S* are based on *F*^2^, conventional *R*-factors *R* are based on *F*, with *F* set to zero for negative *F*^2^. The threshold expression of *F*^2^ > σ(*F*^2^) is used only for calculating *R*-factors(gt) etc. and is not relevant to the choice of reflections for refinement. *R*-factors based on *F*^2^ are statistically about twice as large as those based on *F*, and *R*- factors based on ALL data will be even larger.

### Fractional atomic coordinates and isotropic or equivalent isotropic displacement parameters (Å^2^)

**Table:**

	*x*	*y*	*z*	*U*_iso_*/*U*_eq_	Occ. (<1)
O1	0.26290 (19)	0.81506 (13)	−0.25518 (8)	0.0701 (5)	
O2	0.46504 (19)	0.94889 (13)	−0.21805 (8)	0.0677 (5)	
O3	0.57350 (17)	0.81575 (11)	0.02537 (8)	0.0560 (4)	
O4	0.2953 (2)	0.57310 (12)	0.10823 (8)	0.0769 (6)	
O5	0.29543 (19)	0.29369 (11)	0.23732 (8)	0.0618 (4)	
O6	−0.0235 (2)	0.00816 (12)	0.17156 (9)	0.0732 (5)	
O7	−0.11209 (18)	0.08047 (12)	0.04346 (8)	0.0627 (4)	
C1	0.3099 (2)	0.74316 (15)	−0.13474 (11)	0.0458 (5)	
H1A	0.2355	0.6960	−0.1483	0.055*	
C2	0.3373 (2)	0.81086 (16)	−0.18504 (11)	0.0481 (5)	
C3	0.4469 (2)	0.88311 (16)	−0.16507 (11)	0.0483 (5)	
C4	0.5267 (2)	0.88492 (15)	−0.09551 (11)	0.0456 (5)	
H4A	0.6001	0.9328	−0.0823	0.055*	
C5	0.4985 (2)	0.81575 (14)	−0.04479 (10)	0.0418 (4)	
C6	0.3896 (2)	0.74210 (14)	−0.06348 (10)	0.0405 (4)	
C7	0.3571 (2)	0.67221 (14)	−0.00909 (10)	0.0414 (4)	
H7A	0.3853	0.6931	0.0385	0.050*	
C8	0.2925 (2)	0.58240 (14)	−0.01745 (10)	0.0413 (4)	
C9	0.2669 (2)	0.53078 (14)	0.04976 (10)	0.0441 (5)	
C10	0.2049 (2)	0.42752 (14)	0.04458 (10)	0.0390 (4)	
C11	0.1613 (3)	0.38201 (15)	−0.02935 (10)	0.0502 (5)	
H11A	0.1699	0.3105	−0.0253	0.060*	0.871 (6)
H11B	0.0577	0.3979	−0.0485	0.060*	0.871 (6)
H11C	0.0364	0.3580	−0.0455	0.060*	0.129 (6)
H11D	0.2383	0.3151	−0.0082	0.060*	0.129 (6)
C12A	0.2580 (3)	0.41870 (14)	−0.08081 (12)	0.0498 (8)	0.871 (6)
H12A	0.3610	0.3997	−0.0632	0.060*	0.871 (6)
H12B	0.2261	0.3881	−0.1277	0.060*	0.871 (6)
C12B	0.1315 (14)	0.4496 (10)	−0.0939 (6)	0.082 (8)	0.129 (6)
H12C	0.1311	0.4113	−0.1375	0.098*	0.129 (6)
H12D	0.0337	0.4797	−0.0970	0.098*	0.129 (6)
C13	0.2482 (3)	0.52947 (15)	−0.08856 (11)	0.0575 (6)	
H13A	0.1466	0.5477	−0.1098	0.069*	0.871 (6)
H13B	0.3130	0.5510	−0.1212	0.069*	0.871 (6)
H13C	0.3357	0.4916	−0.0669	0.069*	0.129 (6)
H13D	0.2519	0.5469	−0.1344	0.069*	0.129 (6)
C14	0.1980 (2)	0.38183 (14)	0.10713 (11)	0.0437 (5)	
H14A	0.2338	0.4193	0.1481	0.052*	
C15	0.1438 (2)	0.28305 (14)	0.12215 (10)	0.0395 (4)	
C16	0.1928 (2)	0.24048 (14)	0.19020 (10)	0.0423 (4)	
C17	0.1381 (2)	0.14907 (15)	0.20811 (11)	0.0469 (5)	
H17A	0.1707	0.1224	0.2538	0.056*	
C18	0.0359 (2)	0.09808 (14)	0.15832 (11)	0.0464 (5)	
C19	−0.0115 (2)	0.13695 (15)	0.08948 (11)	0.0439 (5)	
C20	0.0398 (2)	0.22886 (14)	0.07311 (11)	0.0433 (5)	
H20A	0.0041	0.2559	0.0278	0.052*	
C21	0.1569 (3)	0.74047 (18)	−0.27866 (12)	0.0603 (6)	
H21A	0.1141	0.7511	−0.3285	0.090*	
H21B	0.0794	0.7426	−0.2502	0.090*	
H21C	0.2049	0.6769	−0.2732	0.090*	
C22	0.5693 (3)	1.02662 (17)	−0.20014 (14)	0.0640 (7)	
H22A	0.5703	1.0672	−0.2419	0.096*	
H22B	0.6671	0.9993	−0.1840	0.096*	
H22C	0.5414	1.0662	−0.1623	0.096*	
C23	0.6873 (3)	0.88797 (17)	0.04552 (12)	0.0570 (6)	
H23A	0.7343	0.8776	0.0949	0.086*	
H23B	0.6439	0.9530	0.0404	0.086*	
H23C	0.7606	0.8820	0.0149	0.086*	
C24	0.3591 (3)	0.2507 (2)	0.30446 (12)	0.0772 (8)	
H24A	0.4324	0.2950	0.3304	0.116*	
H24B	0.4060	0.1891	0.2963	0.116*	
H24C	0.2821	0.2389	0.3322	0.116*	
C25	−0.0341 (3)	−0.01589 (18)	0.24289 (13)	0.0663 (7)	
H25A	−0.0779	−0.0805	0.2441	0.099*	
H25B	−0.0955	0.0322	0.2612	0.099*	
H25C	0.0640	−0.0159	0.2722	0.099*	
C26	−0.1422 (3)	0.10874 (19)	−0.02995 (12)	0.0634 (7)	
H26A	−0.2049	0.0598	−0.0575	0.095*	
H26B	−0.0500	0.1139	−0.0477	0.095*	
H26C	−0.1923	0.1717	−0.0346	0.095*	

### Atomic displacement parameters (Å^2^)

**Table:**

	*U*^11^	*U*^22^	*U*^33^	*U*^12^	*U*^13^	*U*^23^
O1	0.0786 (11)	0.0790 (12)	0.0460 (9)	−0.0247 (9)	−0.0067 (8)	0.0191 (8)
O2	0.0777 (11)	0.0669 (11)	0.0557 (10)	−0.0219 (9)	0.0045 (8)	0.0270 (8)
O3	0.0667 (10)	0.0512 (9)	0.0453 (8)	−0.0205 (7)	−0.0026 (7)	0.0099 (7)
O4	0.1479 (17)	0.0475 (9)	0.0382 (9)	−0.0341 (10)	0.0246 (9)	−0.0076 (7)
O5	0.0905 (12)	0.0490 (9)	0.0402 (8)	−0.0184 (8)	−0.0030 (8)	0.0028 (7)
O6	0.1228 (15)	0.0437 (9)	0.0548 (10)	−0.0316 (9)	0.0207 (10)	0.0047 (7)
O7	0.0733 (10)	0.0562 (10)	0.0541 (9)	−0.0266 (8)	−0.0009 (8)	0.0056 (7)
C1	0.0488 (11)	0.0429 (11)	0.0450 (11)	−0.0095 (9)	0.0067 (9)	0.0055 (9)
C2	0.0509 (12)	0.0528 (13)	0.0392 (11)	−0.0045 (10)	0.0044 (9)	0.0088 (9)
C3	0.0524 (12)	0.0465 (12)	0.0470 (12)	−0.0039 (10)	0.0117 (9)	0.0142 (9)
C4	0.0463 (11)	0.0394 (11)	0.0506 (12)	−0.0083 (9)	0.0077 (9)	0.0085 (9)
C5	0.0467 (11)	0.0365 (10)	0.0415 (11)	−0.0021 (8)	0.0064 (8)	0.0047 (8)
C6	0.0470 (10)	0.0355 (10)	0.0398 (10)	−0.0018 (8)	0.0098 (8)	0.0056 (8)
C7	0.0506 (11)	0.0367 (10)	0.0367 (10)	−0.0028 (9)	0.0071 (8)	0.0029 (8)
C8	0.0546 (11)	0.0352 (10)	0.0358 (10)	−0.0046 (9)	0.0129 (8)	0.0015 (8)
C9	0.0636 (13)	0.0346 (10)	0.0358 (10)	−0.0067 (9)	0.0133 (9)	0.0000 (8)
C10	0.0484 (11)	0.0318 (10)	0.0381 (10)	−0.0015 (8)	0.0111 (8)	0.0002 (8)
C11	0.0741 (14)	0.0354 (11)	0.0415 (11)	−0.0105 (10)	0.0110 (10)	−0.0025 (9)
C12A	0.0751 (19)	0.0390 (14)	0.0384 (13)	−0.0028 (12)	0.0182 (12)	−0.0078 (10)
C12B	0.11 (2)	0.081 (16)	0.062 (13)	0.035 (14)	0.026 (12)	0.000 (11)
C13	0.0890 (17)	0.0492 (13)	0.0369 (11)	−0.0140 (12)	0.0186 (11)	−0.0012 (9)
C14	0.0568 (12)	0.0356 (10)	0.0392 (10)	−0.0073 (9)	0.0098 (9)	−0.0004 (8)
C15	0.0485 (11)	0.0328 (10)	0.0391 (10)	−0.0014 (8)	0.0133 (8)	0.0027 (8)
C16	0.0551 (12)	0.0354 (10)	0.0380 (10)	−0.0029 (9)	0.0129 (8)	−0.0020 (8)
C17	0.0694 (14)	0.0356 (11)	0.0367 (10)	0.0006 (10)	0.0126 (9)	0.0051 (8)
C18	0.0645 (13)	0.0316 (10)	0.0461 (11)	−0.0049 (9)	0.0179 (10)	0.0032 (8)
C19	0.0488 (11)	0.0376 (11)	0.0456 (11)	−0.0069 (9)	0.0093 (9)	0.0002 (9)
C20	0.0484 (11)	0.0404 (11)	0.0410 (11)	−0.0026 (9)	0.0074 (8)	0.0075 (8)
C21	0.0647 (14)	0.0613 (15)	0.0513 (13)	−0.0012 (12)	0.0007 (11)	−0.0027 (11)
C22	0.0683 (15)	0.0491 (14)	0.0767 (17)	−0.0051 (12)	0.0186 (13)	0.0241 (12)
C23	0.0634 (14)	0.0538 (13)	0.0512 (13)	−0.0166 (11)	0.0030 (10)	−0.0013 (10)
C24	0.113 (2)	0.0700 (17)	0.0398 (13)	−0.0172 (16)	−0.0096 (13)	0.0031 (12)
C25	0.0842 (17)	0.0513 (14)	0.0646 (15)	−0.0113 (13)	0.0168 (13)	0.0208 (12)
C26	0.0717 (16)	0.0591 (15)	0.0535 (14)	−0.0157 (12)	−0.0048 (11)	0.0007 (11)

### Geometric parameters (Å, °)

**Table:**

O1—C2	1.372 (2)	C12A—H12B	0.9700
O1—C21	1.408 (3)	C12A—H13C	1.2119
O2—C3	1.367 (2)	C12B—C13	1.504 (2)
O2—C22	1.413 (3)	C12B—H12C	0.9700
O3—C5	1.372 (2)	C12B—H12D	0.9700
O3—C23	1.422 (2)	C13—H13A	0.9700
O4—C9	1.225 (2)	C13—H13B	0.9700
O5—C16	1.369 (2)	C13—H13C	0.9715
O5—C24	1.416 (3)	C13—H13D	0.9016
O6—C18	1.369 (2)	C14—C15	1.467 (3)
O6—C25	1.402 (3)	C14—H14A	0.9300
O7—C19	1.372 (2)	C15—C16	1.401 (3)
O7—C26	1.412 (3)	C15—C20	1.402 (3)
C1—C2	1.371 (3)	C16—C17	1.394 (3)
C1—C6	1.405 (3)	C17—C18	1.379 (3)
C1—H1A	0.9300	C17—H17A	0.9300
C2—C3	1.396 (3)	C18—C19	1.392 (3)
C3—C4	1.378 (3)	C19—C20	1.380 (3)
C4—C5	1.392 (3)	C20—H20A	0.9300
C4—H4A	0.9300	C21—H21A	0.9600
C5—C6	1.402 (3)	C21—H21B	0.9600
C6—C7	1.462 (3)	C21—H21C	0.9600
C7—C8	1.344 (3)	C22—H22A	0.9600
C7—H7A	0.9300	C22—H22B	0.9600
C8—C9	1.499 (3)	C22—H22C	0.9600
C8—C13	1.506 (3)	C23—H23A	0.9600
C9—C10	1.500 (3)	C23—H23B	0.9600
C10—C14	1.341 (3)	C23—H23C	0.9600
C10—C11	1.507 (3)	C24—H24A	0.9600
C11—C12B	1.504 (2)	C24—H24B	0.9600
C11—C12A	1.505 (2)	C24—H24C	0.9600
C11—H11A	0.9700	C25—H25A	0.9600
C11—H11B	0.9700	C25—H25B	0.9600
C11—H11C	1.1673	C25—H25C	0.9600
C11—H11D	1.1672	C26—H26A	0.9600
C12A—C13	1.503 (2)	C26—H26B	0.9600
C12A—H12A	0.9700	C26—H26C	0.9600
			
C2—O1—C21	117.58 (17)	C12B—C13—H13A	62.9
C3—O2—C22	118.17 (17)	C8—C13—H13A	109.1
C5—O3—C23	117.86 (16)	C12A—C13—H13B	109.1
C16—O5—C24	118.96 (17)	C12B—C13—H13B	132.5
C18—O6—C25	118.51 (18)	C8—C13—H13B	109.1
C19—O7—C26	117.20 (16)	H13A—C13—H13B	107.8
C2—C1—C6	122.64 (19)	C12A—C13—H13C	53.6
C2—C1—H1A	118.7	C12B—C13—H13C	99.5
C6—C1—H1A	118.7	C8—C13—H13C	79.0
C1—C2—O1	124.94 (19)	H13A—C13—H13C	162.5
C1—C2—C3	119.33 (18)	H13B—C13—H13C	83.0
O1—C2—C3	115.72 (18)	C12A—C13—H13D	109.9
O2—C3—C4	124.73 (19)	C12B—C13—H13D	105.8
O2—C3—C2	115.56 (18)	C8—C13—H13D	132.7
C4—C3—C2	119.71 (18)	H13A—C13—H13D	74.8
C3—C4—C5	120.64 (19)	H13C—C13—H13D	111.7
C3—C4—H4A	119.7	C10—C14—C15	131.17 (18)
C5—C4—H4A	119.7	C10—C14—H14A	114.4
O3—C5—C4	122.63 (17)	C15—C14—H14A	114.4
O3—C5—C6	116.53 (16)	C16—C15—C20	116.95 (17)
C4—C5—C6	120.84 (18)	C16—C15—C14	119.13 (17)
C5—C6—C1	116.83 (17)	C20—C15—C14	123.87 (17)
C5—C6—C7	120.34 (17)	O5—C16—C17	122.59 (18)
C1—C6—C7	122.73 (17)	O5—C16—C15	116.35 (17)
C8—C7—C6	129.79 (18)	C17—C16—C15	121.05 (18)
C8—C7—H7A	115.1	C18—C17—C16	120.22 (18)
C6—C7—H7A	115.1	C18—C17—H17A	119.9
C7—C8—C9	116.86 (17)	C16—C17—H17A	119.9
C7—C8—C13	124.92 (17)	O6—C18—C17	123.93 (19)
C9—C8—C13	118.21 (16)	O6—C18—C19	115.96 (18)
O4—C9—C8	120.15 (17)	C17—C18—C19	120.11 (18)
O4—C9—C10	120.45 (17)	O7—C19—C20	124.91 (18)
C8—C9—C10	119.39 (16)	O7—C19—C18	115.92 (17)
C14—C10—C9	116.54 (17)	C20—C19—C18	119.12 (18)
C14—C10—C11	125.28 (17)	C19—C20—C15	122.47 (18)
C9—C10—C11	118.13 (16)	C19—C20—H20A	118.8
C12B—C11—C12A	47.3 (6)	C15—C20—H20A	118.8
C12B—C11—C10	118.5 (6)	O1—C21—H21A	109.5
C12A—C11—C10	111.96 (17)	O1—C21—H21B	109.5
C12B—C11—H11A	131.8	H21A—C21—H21B	109.5
C12A—C11—H11A	109.2	O1—C21—H21C	109.5
C10—C11—H11A	109.2	H21A—C21—H21C	109.5
C12B—C11—H11B	62.9	H21B—C21—H21C	109.5
C12A—C11—H11B	109.2	O2—C22—H22A	109.5
C10—C11—H11B	109.2	O2—C22—H22B	109.5
H11A—C11—H11B	107.9	H22A—C22—H22B	109.5
C12B—C11—H11C	86.2	O2—C22—H22C	109.5
C12A—C11—H11C	125.3	H22A—C22—H22C	109.5
C10—C11—H11C	115.7	H22B—C22—H22C	109.5
H11A—C11—H11C	78.7	O3—C23—H23A	109.5
C12B—C11—H11D	138.9	O3—C23—H23B	109.5
C12A—C11—H11D	95.1	H23A—C23—H23B	109.5
C10—C11—H11D	87.6	O3—C23—H23C	109.5
H11B—C11—H11D	141.2	H23A—C23—H23C	109.5
H11C—C11—H11D	111.6	H23B—C23—H23C	109.5
C13—C12A—C11	111.0 (2)	O5—C24—H24A	109.5
C13—C12A—H12A	109.4	O5—C24—H24B	109.5
C11—C12A—H12A	109.4	H24A—C24—H24B	109.5
C13—C12A—H12B	109.4	O5—C24—H24C	109.5
C11—C12A—H12B	109.4	H24A—C24—H24C	109.5
H12A—C12A—H12B	108.0	H24B—C24—H24C	109.5
C11—C12A—H13C	120.9	O6—C25—H25A	109.5
H12A—C12A—H13C	69.6	O6—C25—H25B	109.5
H12B—C12A—H13C	127.5	H25A—C25—H25B	109.5
C13—C12B—C11	111.1 (2)	O6—C25—H25C	109.5
C13—C12B—H12C	109.4	H25A—C25—H25C	109.5
C11—C12B—H12C	109.4	H25B—C25—H25C	109.5
C13—C12B—H12D	109.4	O7—C26—H26A	109.5
C11—C12B—H12D	109.4	O7—C26—H26B	109.5
H12C—C12B—H12D	108.0	H26A—C26—H26B	109.5
C12A—C13—C12B	47.3 (6)	O7—C26—H26C	109.5
C12A—C13—C8	112.57 (17)	H26A—C26—H26C	109.5
C12B—C13—C8	118.1 (6)	H26B—C26—H26C	109.5
C12A—C13—H13A	109.1		
			
C6—C1—C2—O1	180.0 (2)	C12B—C11—C12A—C13	50.4 (7)
C6—C1—C2—C3	−1.1 (3)	C10—C11—C12A—C13	−58.8 (3)
C21—O1—C2—C1	−4.3 (3)	C12A—C11—C12B—C13	−50.4 (7)
C21—O1—C2—C3	176.8 (2)	C10—C11—C12B—C13	44.1 (15)
C22—O2—C3—C4	−2.2 (3)	C11—C12A—C13—C12B	−50.4 (7)
C22—O2—C3—C2	177.0 (2)	C11—C12A—C13—C8	57.7 (3)
C1—C2—C3—O2	−178.7 (2)	C11—C12B—C13—C12A	50.4 (7)
O1—C2—C3—O2	0.3 (3)	C11—C12B—C13—C8	−45.3 (15)
C1—C2—C3—C4	0.6 (3)	C7—C8—C13—C12A	149.0 (2)
O1—C2—C3—C4	179.6 (2)	C9—C8—C13—C12A	−29.3 (3)
O2—C3—C4—C5	178.8 (2)	C7—C8—C13—C12B	−158.6 (7)
C2—C3—C4—C5	−0.4 (3)	C9—C8—C13—C12B	23.1 (7)
C23—O3—C5—C4	−1.9 (3)	C9—C10—C14—C15	179.5 (2)
C23—O3—C5—C6	178.41 (19)	C11—C10—C14—C15	−3.1 (4)
C3—C4—C5—O3	−178.9 (2)	C10—C14—C15—C16	158.9 (2)
C3—C4—C5—C6	0.7 (3)	C10—C14—C15—C20	−23.7 (3)
O3—C5—C6—C1	178.49 (18)	C24—O5—C16—C17	5.9 (3)
C4—C5—C6—C1	−1.2 (3)	C24—O5—C16—C15	−174.4 (2)
O3—C5—C6—C7	2.0 (3)	C20—C15—C16—O5	179.37 (18)
C4—C5—C6—C7	−177.69 (19)	C14—C15—C16—O5	−3.1 (3)
C2—C1—C6—C5	1.4 (3)	C20—C15—C16—C17	−0.9 (3)
C2—C1—C6—C7	177.8 (2)	C14—C15—C16—C17	176.60 (19)
C5—C6—C7—C8	−159.1 (2)	O5—C16—C17—C18	−179.4 (2)
C1—C6—C7—C8	24.6 (3)	C15—C16—C17—C18	1.0 (3)
C6—C7—C8—C9	−176.7 (2)	C25—O6—C18—C17	27.4 (3)
C6—C7—C8—C13	5.0 (4)	C25—O6—C18—C19	−153.5 (2)
C7—C8—C9—O4	4.8 (3)	C16—C17—C18—O6	−179.9 (2)
C13—C8—C9—O4	−176.8 (2)	C16—C17—C18—C19	1.0 (3)
C7—C8—C9—C10	−175.86 (18)	C26—O7—C19—C20	13.3 (3)
C13—C8—C9—C10	2.6 (3)	C26—O7—C19—C18	−169.2 (2)
O4—C9—C10—C14	−6.9 (3)	O6—C18—C19—O7	0.2 (3)
C8—C9—C10—C14	173.78 (19)	C17—C18—C19—O7	179.32 (19)
O4—C9—C10—C11	175.5 (2)	O6—C18—C19—C20	177.82 (19)
C8—C9—C10—C11	−3.9 (3)	C17—C18—C19—C20	−3.0 (3)
C14—C10—C11—C12B	162.0 (7)	O7—C19—C20—C15	−179.5 (2)
C9—C10—C11—C12B	−20.6 (7)	C18—C19—C20—C15	3.1 (3)
C14—C10—C11—C12A	−145.8 (2)	C16—C15—C20—C19	−1.1 (3)
C9—C10—C11—C12A	31.6 (3)	C14—C15—C20—C19	−178.53 (19)

### Hydrogen-bond geometry (Å, °)

**Table:**

*D*—H···*A*	*D*—H	H···*A*	*D*···*A*	*D*—H···*A*
C12A—H12B···O1^i^	0.97	2.43	3.364 (3)	160
C25—H25B···O1^ii^	0.96	2.55	3.450 (3)	156

Symmetry codes: (i) −*x*+1/2, *y*−1/2, −*z*−1/2; (ii) −*x*, −*y*+1, −*z*.
